# Alpha-Arbutin Promotes Wound Healing by Lowering ROS and Upregulating Insulin/IGF-1 Pathway in Human Dermal Fibroblast

**DOI:** 10.3389/fphys.2020.586843

**Published:** 2020-11-04

**Authors:** Natalia Polouliakh, Vanessa Ludwig, Akira Meguro, Tatsukata Kawagoe, Oliver Heeb, Nobuhisa Mizuki

**Affiliations:** ^1^Department of Ophthalmology and Visual Sciences, Yokohama City University Graduate School of Medicine, Yokohama, Japan; ^2^Sony Computer Science Laboratories Inc., Tokyo, Japan; ^3^Scientista Co., Ltd., Tokyo, Japan; ^4^Department of Biology, ETH Zürich, Zurich, Switzerland; ^5^Department of MAVT, ETH Zürich, Zurich, Switzerland

**Keywords:** alpha-arbutin, gene expression, phylogenetic footprinting, anti-oxidative activities, Nrf2-signaling

## Abstract

Alpha-arbutin (4-hydroxyphenyl alpha-glucopyranoside) is a known inhibitor of tyrosinase in keratinocytes; however, its effect on other genes and pathways in other skin cells has not been thoroughly investigated. In this study, we investigate the mechanism of alpha-arbutin activity in human dermal fibroblast cultures for 48 h. Results showed that the oxidative stress pathway was activated as alpha-arbutin reduced reactive oxygen species. In addition, we found a high possibility of wound healing and the upregulation of the insulin-like growth factor 1 receptor (IFG1R) pathway. We also investigated the role of the NRF2 gene in mediating the alpha-arbutin response. *In silico* comparative genomics analysis conducted using our original tool, SHOE, suggested transcription factors with a role in tumor suppression and toxicity response as candidates for regulating the alpha-arbutin–mediated pathway.

## Introduction

Skin aging is largely caused by extrinsic factors, namely, continual exposure to oxidative environmental stimuli such as solar radiation, cigarette smoke, and other pollutants (Cho et al., 2009). Constant exposure to solar radiation leads to chronic transdermal water loss, atopic dermatitis, psoriasis, and skin cancer (Skobowiat et al., 2013; Bebeshko et al., 2017). The second largest contributor to skin aging is an intrinsic factor, the age-related mitochondrial enzyme dysfunction that inhibits epidermal regeneration (Quan et al., 2015).

At the molecular level, skin aging is characterized by reduced procollagen synthesis and degradation of the extracellular matrix mainly comprising collagen, glycosaminoglycan, and elastin. Aged skin fibroblasts become detached from the destabilized extracellular matrix, leading to a rounded and collapsed appearance. Furthermore, excessive dryness increases cortisol secretion in the brain, which may induce neurogenerative diseases such as Parkinson and Alzheimer diseases (Dadgar et al., 2018; Ding et al., 2020). Chronic neurogenerative disorders can affect the function of tight junction proteins in the skin (Jin et al., 2016; Sugita et al., 2019).

We recently investigated the effect of chum salmon egg (CSE) extract on collagen synthesis and CSE antiaging activity in human dermal fibroblasts (Yoshino et al., 2016). In this study, we found that supplementing arbutin on human dermal fibroblasts decreased reactive oxygen species (ROS) through activation of the oxidative stress pathway, activated the insulin-like growth factor 1 receptor (IGF1R) pathway, and promoted consequent wound healing by downregulating the matrix metallopeptidases (MMPs) network. The results also revealed that the nuclear receptor factor 2 (NFE2L2) plays a pivotal role in the signaling of the downstream regulation of oxidative stress pathway genes.

## Materials and Methods

### Cell Cultures

Normal human neonatal skin fibroblasts (NB1RGB cells) were purchased from the RIKEN Cell Bank (Ibaraki, Japan). The cells were cultured in alpha-Minimum Essential Medium (MEM) medium (Thermo Fisher Scientific, Waltham, MA, United States) supplemented with 10% fetal bovine serum (Thermo Fisher Scientific), penicillin 100 U/mL, and streptomycin 2.5 μL/mL. Cells were plated at a concentration of 1 × 10^5^ cells/dish (60 mm), and the plates were supplemented with a 1% alpha-arbutin (GLICO Co. Ltd.) solution or distilled water (Gibco) as a negative control. This dose was selected because 1% solution is the maximum permitted dose in over-the-counter cosmetic products. After incubating for 48 h at 37°C in humidified air with 5% CO_2_, the total RNA was isolated from the fibroblasts using the RNeasy kit (Qiagen) according to the manufacturer’s protocol. The extracted RNA was stored at −80°C.

### Gene Expression Assay

A reverse transcription of the total RNA was carried out using the SuperScript II Reverse Transcriptase (Thermo Fisher Scientific), which was stored at 4°C. A quantitative real-time polymerase chain reaction (PCR) of the MMP3, EGFR, FOXO1, SIRT1, COL1A1, IGF1R1, ELOVL3, and NFE2L2 (Nrf2) genes was performed using the StepOnePlus Real-Time PCR System (Thermo Fisher Scientific) with TaqMan gene expression assays. A relative quantification of gene expression was performed using the standard curve method, with glyceraldehyde-3-phosphate dehydrogenase (GAPDH) gene as an endogenous control.

An unpaired statistical *t* test was performed to verify the significance between expression in control and arbutin supplementation condition. Genes were normalized to GAPDH housekeeping genes before performing the *t* test.

### Oxidative Stress Analysis PCR Array

After using RT-PCR to verify changes in gene expression, we assessed the total antioxidant profile of the cells incubated for 48 h using the Human Oxidative Stress RT2 Profiler PCR Array (Qiagen NV), which contains 84 key genes related to the oxidative stress response. All reactions were carried out using the StepOnePlus Real-Time PCR System according to the manufacturer’s protocol and statistical analysis. The relative expression values were calculated using the 2^–ΔΔCt^ method in which the cutoff was set to 35 Ct. Genes with low absolute expression levels were excluded from further analyses. All values from both the TaqMan assay and PCR array experiments are presented as the arithmetic means of two biological replicates.

### Promoter Analysis *in silico*

Phylogenetic footprinting using the SHOE software (Polouliakh et al., 2018) previously developed by the authors was performed on genes overexpressed in the PCR oxidative stress array and gene expression assay. An analysis workflow was performed on the results of SHOE using the REACTOME database (Caley et al., 2015) and CellDesigner pathway editor (Funahashi et al., 2007).

## Results

### Genes Influenced by Alpha-Arbutin

**FIGURE 1:**
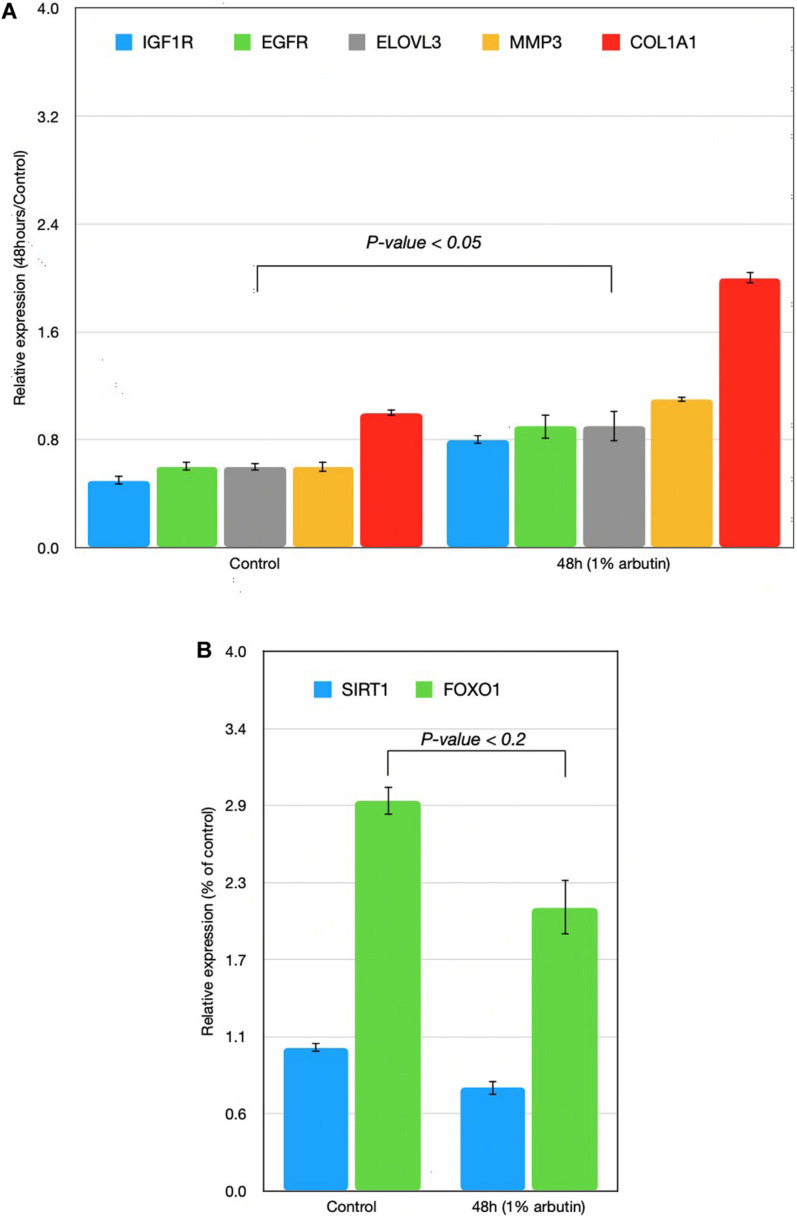
Time-series expressions of seven genes [**(A)** IGF1R, EGFR, ELOVL3, MMP3, COL1A1; **(B)** SIRT1, FOXO1] with 1% arbutin solution after gene expression assay was performed on skin fibroblasts. Results were attained using the 2^–ΔΔCt^ method. The glyceraldehyde-3-phosphate dehydrogenase (GAPDH) gene was used as an endogenous control and was compared with a control group in which the skin fibroblast was supplemented with distilled water.

**FIGURE 2:**
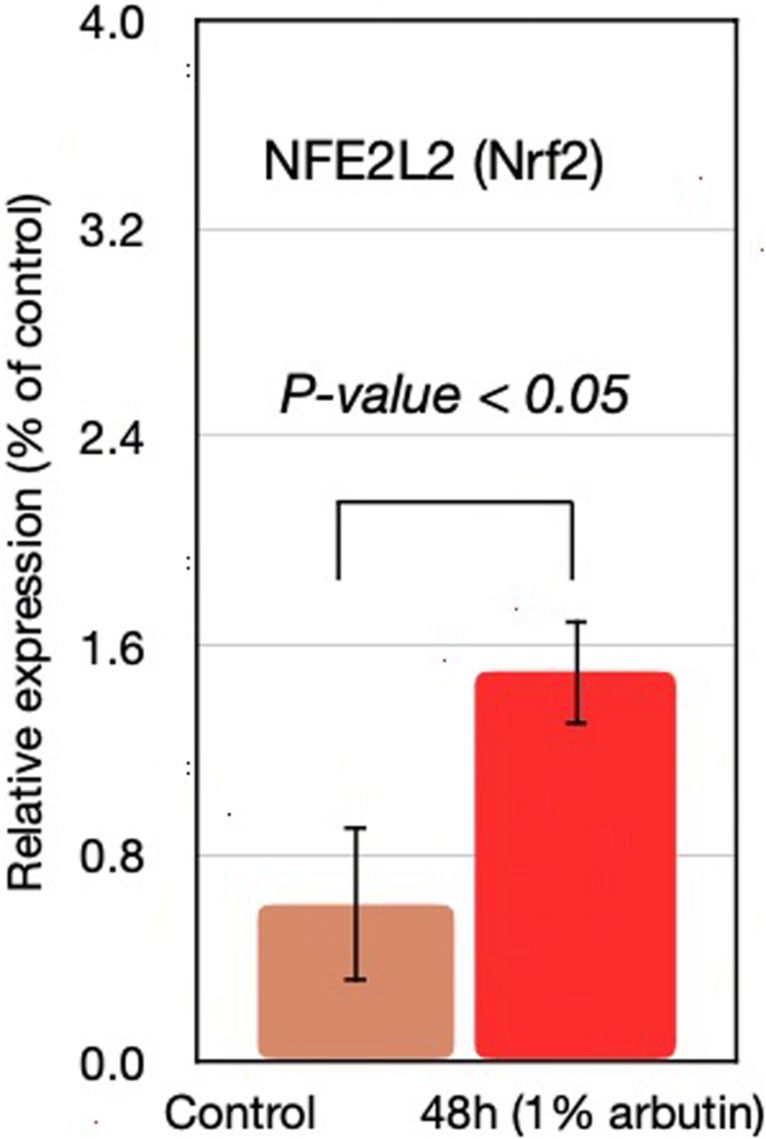
NFE2L2 gene expression at 48 h with supplementation of 1% arbutin solution.

This study represents an initial step toward investigating the beneficial effects of alpha-arbutin on human dermal fibroblast cell cultures. A gene expression assay revealed that treatment with alpha-arbutin enhanced the mRNA expression of type I procollagen (COL1A1), matrix metallopeptidase 3 (MMP3), ELOVL fatty acid elongase 3 (ELOVL3), insulin-like growth factor 1 receptor (IGF1R), and epidermal growth factor receptor (EGFR). On the other hand, forkhead box protein O1 (FOXO1) and sirtuin 1 (SIRT1) were downregulated by the supplementation of alpha-arbutin (Figures 1A,B). The RT2 Profiler PCR Array analysis of the oxidative stress pathway revealed 34 genes that doubled or halved their expression, and among them, the following 11 genes are involved in the downstream regulation of NFE2L2 (Nrf2) gene (Table 1) upon exposure to hydroquinone. They are TXN, GSTP1, TXNRD1, SQSTM1, PRDX4, GPX3, GSTZ1, NQO1, HMOX1, GCLM, and FTH1 genes. NFE2L2 has also been reported to be activated by hydroquinone and tert-butylhydroquinone (Papaconstantinou, 2009). Arbutin may release hydroquinone after its glycosidic bonds are cleaved or through hydrolysis by human skin bacteria (Tada et al., 2014). The arbutin and hydroquinone structures are shown in Supplementary Figure 1. The only difference between the alpha-arbutin and hydroquinone structures is the glycosidic bonds (sugar chain). In our study, NFE2L2 was upregulated at 48 h (Figure 2). From this, we concluded that alpha-arbutin may activate NFE2L2, which consequently activates target genes that reduce ROS.

**TABLE 1:** Differentially regulated genes involved in oxidative resistance and reactive oxygen species metabolism after performing PCR array analysis.

Gene	Description	Fold change	
		Exp 1 (passage 7)	Exp 2 (passage 11)
*DHCR24*	24-Dehydrocholesterol Reductase	4.77	2.84
*SIRT2*	Sirtuin 2	4.76	1.88
*TXNRD1*	Thioredoxin Reductase 1	4.73	2.88
*HMOX1*	Heme Oxygenase 1	4.64	3.47
*UCP2*	Uncoupling Protein 2	2.43	2.88
*PNKP*	Polynucleotide Kinase 3’-Phosphatase	2.41	2.88
*NQO1*	NAD(P)H Quinone Dehydrogenase 1	2.40	2.42
*PTGS2*	Prostaglandin-Endoperoxide Synthase 2	2.40	1.42
*SQSTM1*	Sequestosome 1	2.39	1.71
*GPX3*	Glutathione Peroxidase 3	2.39	2.13
*HSPA1A*	Heat Shock Protein Family A (Hsp70) Member 1A	2.38	2.28
*RNF7*	Ring Finger Protein 7	2.38	1.48
*GSTP1*	Glutathione S-Transferase Pi 1	2.38	2.80
*GCLM*	Glutamate-Cysteine Ligase Modifier Subunit	2.38	2.55
*TXN*	Thioredoxin	2.38	2.38
*FTH1*	Ferritin Heavy Chain 1	2.38	2.21
*GSTZ1*	Glutathione S-Transferase Zeta 1	2.37	2.81
*PRDX5*	Peroxiredoxin 5	2.37	2.81
*SRXN1*	Sulfiredoxin 1	2.37	1.40
*GSS*	Glutathione Synthetase	2.37	1.91
*CCS*	C-C Motif Chemokine Receptor 5	2.37	2.83
*FOXM1*	Forkhead Box M1	2.36	11.23
*PRDX4*	Peroxiredoxin 4	2.35	2.85
*SOD3*	Extracallular superoxide dismutaze [Cu-Zn]	2.30	2.86
*TXNRD2*	Thioredoxin Reductase 2	2.30	2.86
*COL1A1*	Collagen 1 A 1	2.10	1.81
*GPX1*	Glutathione peroxidase 1	1.45	1.53
*NOS2*	Nitric Oxide Synthase 2	−2.94	−2.55
*LPO*	Lactoperoxidase	−2.97	−2.30
*NOX5*	NADPH Oxidase 5	−3.23	−2.71
*EPX*	Eosinophil Peroxidase	−3.04	−2.58
*DUOX2*	Dual Oxidase 2	−3.10	−2.93
*APOE*	Apolipoprotein E	−3.26	−1.24
*SEPP1*	Selenoprotein P	−3.54	−1.26

*The fold change threshold was set to ±2 to define significant increases or decreases in gene expression (indicated in bold). Analysis was performed in two passages (experiments 1 and 2).*

The upregulated genes in the oxidative stress pathway include 24-dehydrocholesterol reductase (DHCR24), sirtuin 2 (SIRT2), thioredoxin reductase 1 (TXNRD1), and heme oxygenase 1 (HMOX1). Members of the peroxiredoxin system (PRDX4, PRDX5) and a member of the glutathione peroxidase system (GPX3) were also upregulated, and they each represent two different systems in the oxidative stress response. The glutathione metabolic pathway was prominently upregulated (GPX3, GSS, GSTP1, GCLM, GSTZ1 genes), and its fold changes ranged from 2.37 to 2.39.

Other noticeably upregulated genes directly or indirectly involved in the antioxidative status of the cell include sulfiredoxin 1 (SRXN1), which works with PRDX4; HSPA1A, which stabilizes proteins against aggregation; NQO1, which reduces antioxidant molecules; and FTH1, which is involved in iron storage. The prominently downregulated genes contained selenoprotein P (SEPP1), dual oxidase 2 (DUOX2), apolipoprotein E (APOE), eosinophil peroxidase (EPX), the NADPH oxidase 5 (NOX5), lactoperoxidase (LPO), and nitric oxide synthase 2 (NOS2).

### Wound Healing and Oxidative Stress Response

**FIGURE 3:**
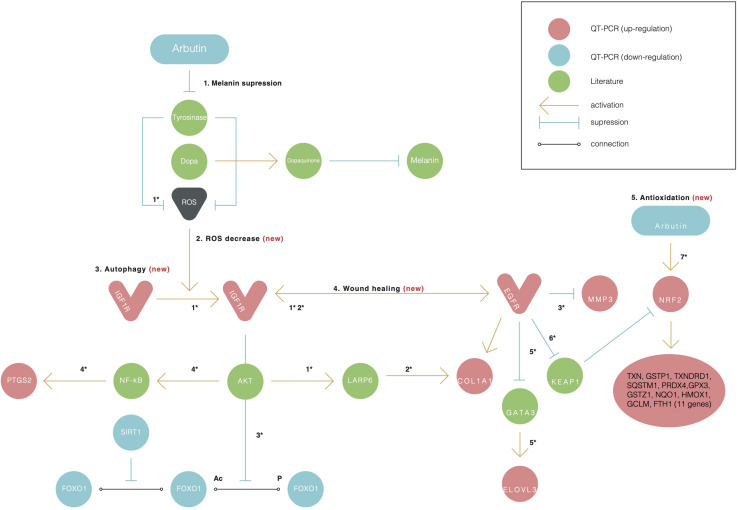
Alpha-arbutin regulation map showing the downstream regulation of genes starting with alpha-arbutin as the origin. In addition to melanin suppression (1), three other newly identified activities of arbutin such as (2) ROS decrease, (3) autophagy, (4) wound healing, and (5) antioxidation are shown. Numbers with asterisks (*) refer to the reactions discussed in the manuscript. The asterisk (*) refers to the reaction between one gene and another and is discussed further in the discussion with literature referred.

First, we assumed that alpha-arbutin is involved in wound healing and the oxidative stress response. This is because the MMP3 gene regulates wound healing rate through its role in wound contraction, as shown in a previous study (Caley et al., 2015). The upregulation of MMP3 and EGFR was previously identified (Kusukawa et al., 1996) (Figure 3, reaction 1) as well. In our study, we also observed upregulation for EGFR (Figure 1A). The downregulation of FOXO1 at 48 h (Figure 1B) provided further evidence of arbutin’s involvement in wound healing. Enhanced wound repair and reduced scarring were observed in a previous study on FOXO1± mice (Mori et al., 2014), which supports our findings.

The gene expression of type I collagen α1 (COL1A1) was found to significantly decrease at the wound sites of FOXO1± mice 7 days after injury, which is believed to be the reason for less scarring (Caley et al., 2015). COL1A1 was downregulated after 24 h (data not shown) and upregulated after 48 h in our study (Figure 1A).

FOXO1 has been reported to target genes coding for intracellular and extracellular antioxidant proteins and is deacetylated by SIRT1 (Figure 3, reaction 2), which results in its activation in most cases (Kusukawa et al., 1996; Caley et al., 2015). In our findings, SIRT1 was also downregulated, supporting the notion that FOXO1 is inactive. SIRT1 downregulation phosphorylates FOXO1, which is consequently inactivated by the serine/threonine-specific protein kinase B (Akt), as shown in Figure 3, reaction 3. Kinase Akt may be inactivated by stimulating cells with insulin (Caley et al., 2015). Akt is downstream of insulin/IGF-1 signaling and is activated by PI3K through the phosphorylation of insulin receptor substrates-1 and -2 (Kusukawa et al., 1996).

### IGF1R and Insulin Pathway Activation

IGF1R was upregulated in our findings; this further suggests that the insulin pathway is active, meaning that Akt phosphorylates and inactivates FOXO1. EGFR and IGF1R were found to interact on multiple levels in the early stages and activated each other (Figure 3, reaction 2) directly or indirectly (Zellmer et al., 2010; Mori et al., 2014).

The EGFR-mediated repression of GATA3 activated the transcription of ELOVL3 (Sugimoto et al., 2004) (Figure 3, reaction 5), supporting our finding of increased ELOVL3 expression. ELOVL3 is a fatty acid chain elongation enzyme necessary for skin functions. IGF1R was found to regulate LARP6 expression in an Akt signaling–dependent manner. The activation of the pathway led to an upregulation of LARP6, causing an increase in COL1A1 expression (Hughes et al., 2011) (Figure 3, reaction 2). The Ins/IGF-1 signaling pathway itself is of particular interest because of the changes in its responsiveness to the ROS environment; lower levels of ROS activate the pathway, whereas higher ROS levels inhibit its signaling processes (Gross et al., 2009). Alpha-arbutin has been found to inhibit the formation of hydroxyl radicals via L-tyrosine-tyrosinase (Figure 3, reaction 7), and alpha-arbutin is expected to alleviate oxidative stress derived from the melanogenic pathway in the skin (Kousteni, 2011). This supports the notion that the lower ROS levels were a result of the alpha-arbutin treatment and its inhibitory effect on tyrosinase.

### Downregulated Genes and APOE4

PTGS2 was found to be induced by Akt and hypothesized to be active throughout the NF-κB pathway in mutated PTEN endometrial cancer cells (van der Veeken et al., 2009) (Figure 3, reaction 4). It is unclear why NOS and SEPP1, both expressed predominantly in the liver, are downregulated. It is also unclear why NOX5 predominantly expressed in the testis and lymphocyte-rich areas of the spleen and lymph nodes is downregulated in alpha-arbutin–treated fibroblasts. Among the downregulated genes, apolipoprotein 4 (APOE4) is of particular interest (Table 1). It was previously found that the downregulation of APOE4 at a mature age attenuates Parkinson disease (Jin et al., 2016; Sugita et al., 2019); however, its downregulation by alpha-arbutin was unknown.

### *In silico* Transcription Regulation Prediction

**FIGURE 4:**
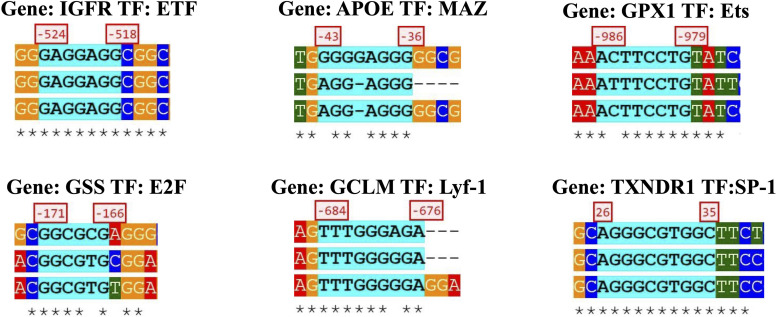
Specific gene sequence from a transcription factor and the orthologous species alignment.

Twenty-eight out of the 42 genes (34-gene PCR array + 8-gene gene assay) analyzed with SHOE had orthologs in mice and rats. Fifteen genes were determined by SHOE as those having cross-species conserved transcription factors binding motifs (Supplementary Table 1). The motifs that matched the consensus sequence to ≥70% were selected for Supplementary Table 1 and sorted accordingly. Examples of the discovered motifs can be seen in Figure 4. Supplementary Figure 2 shows the further analysis workflow after identifying the transcription factor motifs, and Supplementary Figure 3 shows genes visualized by CellDesigner having cross-species conserved transcription factors binding motifs (Kusukawa et al., 1996).

Here, select genes and transcription factor motifs are discussed; the rest can be viewed and downloaded on the SHOE site^1^.

The IGFR1 gene had the most cross-species conserved promoters between human–mouse–rat (85 sites), with top candidate transcription factors such as E2F (2 sites), TFII-I (2 sites), SP1 (6 sites), ETF (10 sites), NF-κB (1 site), and c-Rel (1 site). (Hereafter, the numbers of copies of the site per motif name are indicated by numbers only.)

The second most substantial conserved region was located in the APOE gene (27 sites), with the top motifs MAZ (4), E2F (2), ETF (2), SP1 (4), CAC-binding (2), and ZF5 (2). The third was the GPX1 gene (22 sites), with top-scoring AML1 (2), Ets (3), E2F, AP-1, MAZ, NF-E2, and USF.

The GSS gene (17 sites) had E2F (2), SP1 (3), MAZ, NRF1(2), LF-A1, CAC binding, KROX (4) motifs, and the GCLM gene (15 sites) appeared to have Lyf-1, STATx, SP1, E2F (2), and NFE2L2 motifs in the promoter, which emphasizes our hypothesis of regulation overexpressed genes via NFE2L2.

E2F, ETF, and SP-1 sites were present in nearly all genes. E2F transcription factors are known to regulate many genes involved in DNA synthesis and cell cycle entry (Mori et al., 2014). Together with ETF and SP-1, E2F1 was found to have an essential role in murine hepatocytes in the process of proliferation-dependent differential gene expression (Zellmer et al., 2010). E2F1 and SP1 were previously demonstrated to functionally and physically interact with each other and may regulate the transcription of genes that contain one or both binding sites for the respective transcription factors (Hughes et al., 2011).

## Discussion

This study represents an initial step toward investigating the beneficial effects of alpha-arbutin on human dermal fibroblast cell cultures. Basing on our results (see section *Results*), we assume alpha-arbutin is involved in wound healing and upregulation of the insulin/IGF-1 pathway that indicates anti-inflammatory and antiaging properties of this ingredient. Oxidative stress pathway analysis revealed that the peroxidase system and glutathione metabolic pathway are prominently upregulated by alpha-arbutin. Among genes upregulated, 11 genes overlapped with the NFE2L2(Nrf2)-mediated response induced by hydroquinone supplementation. In our study, NFE2L2 (gene expression assay) was upregulated at 48 h. From this, we assume that alpha-arbutin may activate NFE2L2, which consequently activates target genes that reduce ROS. This possibility is raised by the reason that the only difference between the alpha-arbutin and hydroquinone structures is the glycosidic bonds that can be easily cleaved by physical or bacterial interaction. Alpha-arbutin’s (Sugimoto et al., 2004) inhibitory role on melanogenesis in cultured human melanoma cells, as well as lightening effect on human skin, has already been discussed (Thongchai et al., 2007). Also, anticancer and anti-inflammation activities of alpha-arbutin have been reported. For example, alpha-arbutin protects cells from apoptosis induced by X-irradiation in U937 cells via decreasing intracellular hydroxyl radical production (Wu et al., 2014). Besides, alpha-arbutin inhibits TCCSUP human bladder cancer cell proliferation via the upregulation of p21 (Li et al., 2011). In addition, it is reported that four downregulated genes of AKT1, CLECSF7, FGFR3, and LRP6 served as candidate genes and correlated with suppressing the biological processes in the cell cycle of cancer progression and the downstream signaling pathways of malignancy of melanocytic tumorigenesis (Cheng et al., 2007). In our study, IGF1R was found to regulate LARP6 expression in an Akt signaling–dependent manner. Another study reports that it reduces Bax/Bcl-2 ratio, P53 mRNA expression, and necrosis in fibroblasts exposed to the tert-Butyl hydroperoxide (Ebadollahi et al., 2020). In *in silico* analysis of transcription regulation, we identified several transcription factors involved in toxicity and tumorigenic response mechanism (Polouliakh et al., 2018). Alpha-arbutin anti-inflammation properties in the field of neurogenerative disease and disorders such as Parkinson and Alzheimer diseases (Dadgar et al., 2018; Ding et al., 2020) were observed, and in our study, APOE4 is downregulated too, which highlights the similar response on alpha-arbutin treatment in different cells.

## Conclusion

Our study determined the antioxidative activity of alpha-arbutin to the human dermal fibroblast in 48 h after supplementation. Our study showed that alpha-arbutin enhances the wound healing process in human dermal fibroblasts via activation of the MMP3, EGFR, and COL1A1 genes and suppression of the FOXO1 and SIRT1 genes. We also found that a decrease in ROS activates the Ins/IGF-1 signaling pathway, which is indispensable for the skin autophagic process. Further, 34 genes of the oxidative stress pathway undergo significant change upon alpha-arbutin supplementation, and NFE2L2 (Nrf2) gene is a candidate for mediating its external signal. We believe that the positive effect of alpha-arbutin will offer insights into healthy skin maintenance, which, together with oral supplementation, may be crucial in the treatment and prevention of age-susceptible diseases.

## Data Availability Statement

The raw data supporting the conclusions of this article will be made available by the authors, without undue reservation. Requests to access these datasets should be directed to nata@csl.sony.co.jp.

## Author Contributions

NP and VL conceived the topic and conducted the experiment, contributed equally. AM, TK, and NM supervised the experimental part and conducted the discussion. OH provided technical support for manuscript preparation and review. All authors contributed to the article and approved the submitted version.

## Conflict of Interest

NP is an employee of Sony Computer Science Laboratories, Inc. and also the president and CEO of Scientista Co., Ltd. These companies did not provide funding for this study. Scientista Co., Ltd. sells a cosmetic compounded with alpha-arbutin: however, this situation did not affect the results reported in this study. The remaining authors declare that the research was conducted in the absence of any commercial or financial relationships that could be construed as a potential conflict of interest.

## Footnotes

1 http://ec2-54-150-223-65.ap-northeast-1.compute.amazonaws.com

## Supplementary Material

Supplementary Figure 1 Alpha-arbutin and Hydroquinone structure acquired from Wikipedia.

Supplementary Figure 2 Visualization of the results of SHOE analysis and workflow on REACTOME and CellDesigner software tool of the GPX1 gene, principally the AML-1a transcription factor-binding motif. Note: Upper left, a full list of and the related motifs of arbutin is presented. In addition, the table shows the motif sequence, the consensus sequence, the similarity matching score and as well the pareto number.Upper right, alignment of the orthologous three species of the GPX1 promoter for the motif ALM-1a (H, human; M, mice; R, rat).Below left, the result table from SHOE is transferred to the program CellDesigner and the results are visualized in a hierarchical map.Below right, the program Reactome can also be referred to the data of SHOE and visualizes the pathway.

Supplementary Figure 3 Visualization of 15 orthologous genes using CellDesigner pathway editor.

Supplementary Table 1 Oxidative stress gene with high expression change and a matching score to the consensus sequence over 70% are listed with the related transcription factor. Whereat the motifs from the oxidative stress gene are conserved.The complete list can be found on the SHOE site: http://ec2-54-150-223-65.ap-northeast-1.compute.amazonaws.comAbbreviation: NM, Refseq transcript number; TF, transcription factor.Note: The numbers behind the consensus presents the matching score between the motif and the consensus. In the second brackets, It is shown the pareto number.
