# Corrigendum: Alpha-Arbutin Promotes Wound Healing by Lowering ROS and Upregulating Insulin/IGF-1 Pathway in Human Dermal Fibroblast

**DOI:** 10.3389/fphys.2021.659813

**Published:** 2021-03-12

**Authors:** Natalia Polouliakh, Vanessa Ludwig, Akira Meguro, Tatsukata Kawagoe, Oliver Heeb, Nobuhisa Mizuki

**Affiliations:** ^1^Department of Ophthalmology and Visual Sciences, Yokohama City University Graduate School of Medicine, Yokohama, Japan; ^2^Sony Computer Science Laboratories Inc., Tokyo, Japan; ^3^Scientista Co., Ltd., Tokyo, Japan; ^4^Department of Biology, ETH Zürich, Zurich, Switzerland; ^5^Department of MAVT, ETH Zürich, Zurich, Switzerland

**Keywords:** alpha-arbutin, gene expression, phylogenetic footprinting, anti-oxidative activities, Nrf2-signaling

In the published article, there was an error regarding the affiliations for **Natalia Polouliakh**. As well as having affiliation(s) Department of Ophthalmology and Visual Sciences, Yokohama City University Graduate School of Medicine, Yokohama, Japan and Sony Computer Science Laboratories Inc., Tokyo, Japan, **Natalia Polouliakh** should also have Scientista Co., Ltd., Tokyo, Japan.

In the published article, the Conflict of Interest for Natalia Polouliakh (NP) was not sufficiently disclosed. The Conflict of Interest should have contained the following disclosure:

NP is an employee of Sony Computer Science Laboratories, Inc. and also the president and CEO of Scientista Co., Ltd. These companies did not provide funding for this study. Scientista Co., Ltd. sells a cosmetic compounded with alpha-arbutin: however, this situation did not affect the results reported in this study.

The remaining authors declare that the research was conducted in the absence of any commercial or financial relationships that could be construed as a potential conflict of interest.

The authors apologize for this error and state that this does not change the scientific conclusions of the article in any way. The original article has been updated.

