# Supplementary figures and images for: Alpha-Arbutin Promotes Wound Healing by Lowering ROS and Upregulating Insulin/IGF-1 Pathway in Human Dermal Fibroblast
Source: Front Physiol. 2020 Nov 4;11:586843. doi: 10.3389/fphys.2020.586843 (PMC7672191; doi:10.3389/fphys.2020.586843)

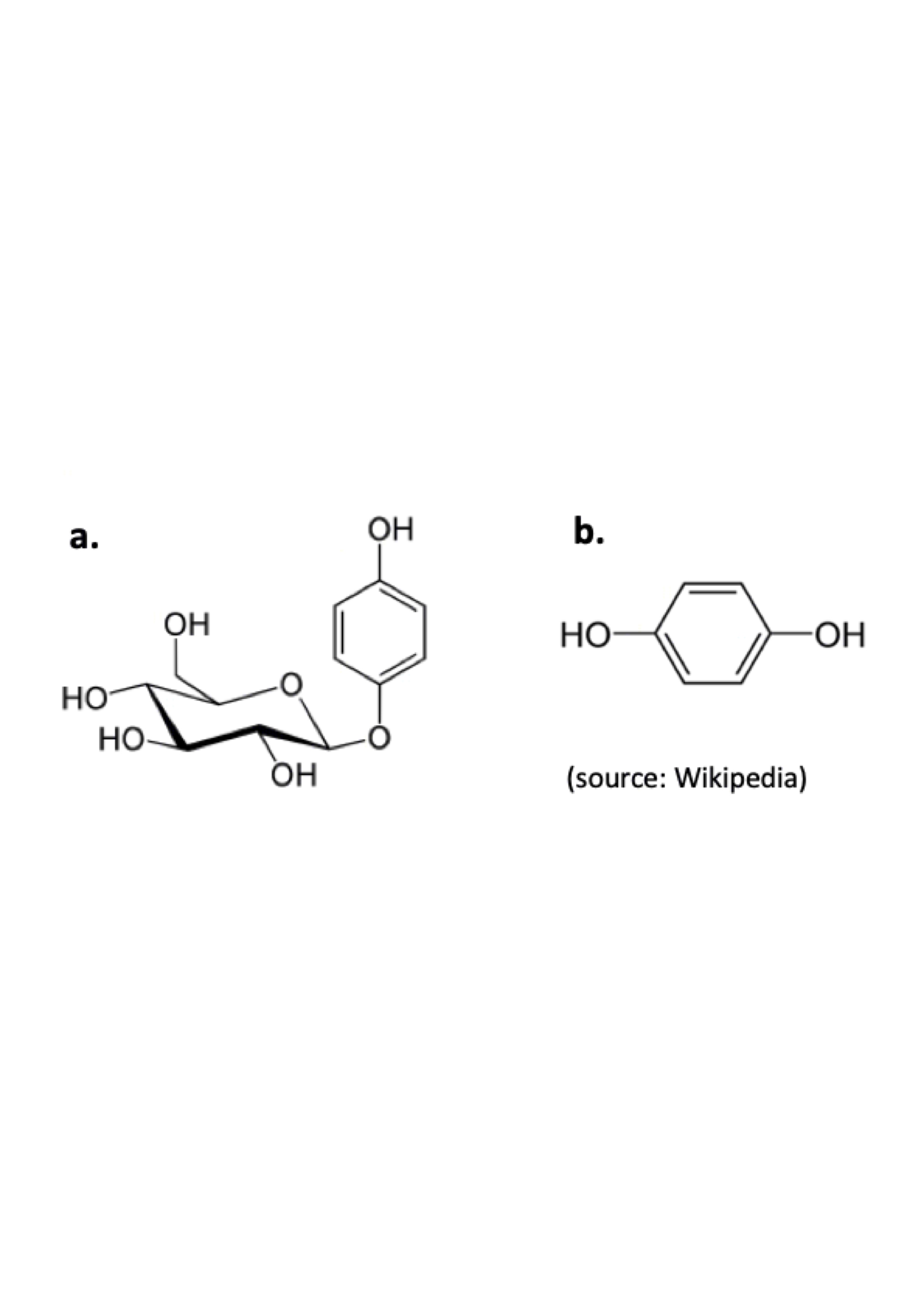

Supplement: Supplementary Figure 1 — Alpha-arbutin and Hydroquinone structure acquired from Wikipedia. [file Image_1.JPEG]

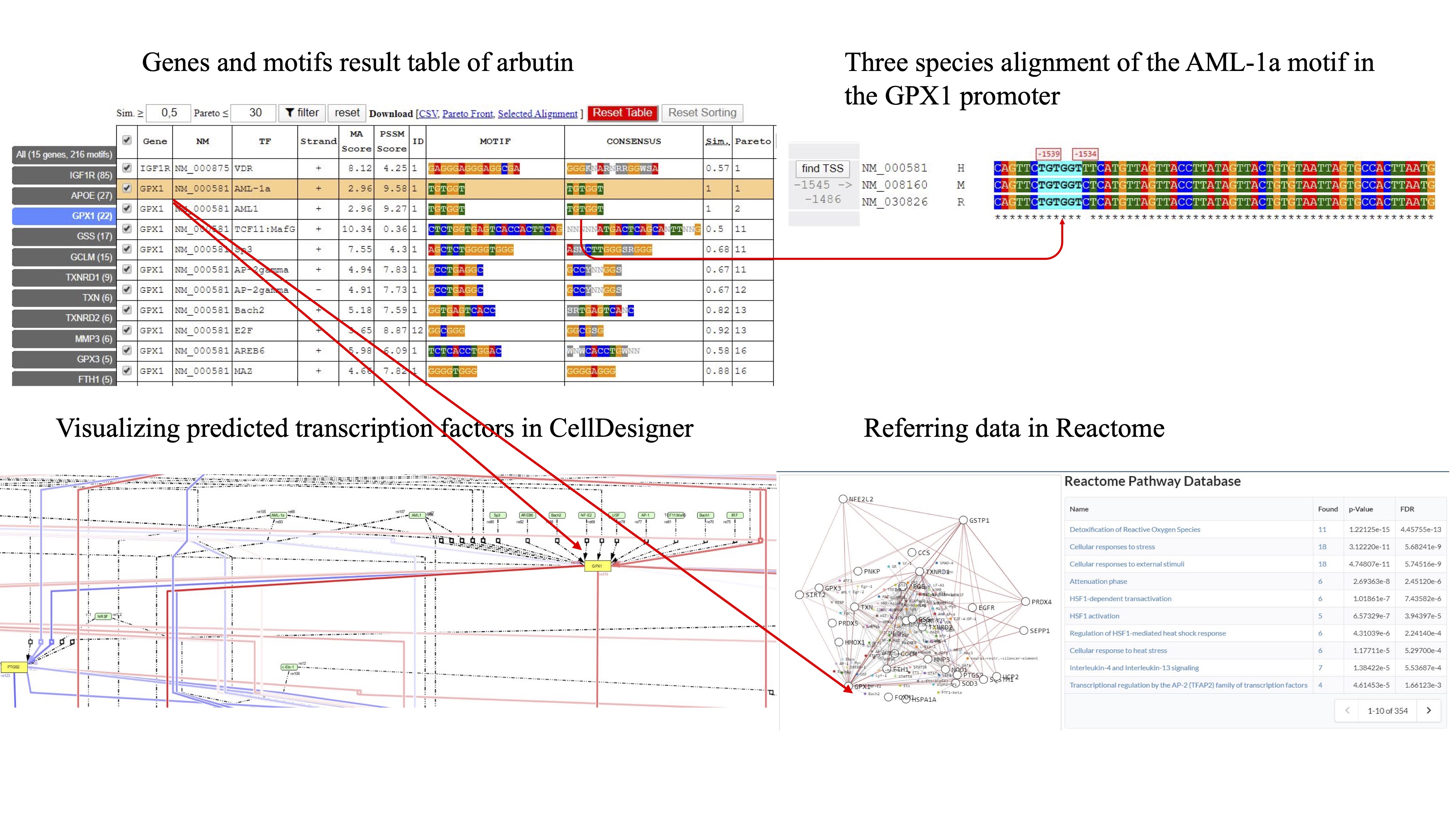

Supplement: Supplementary Figure 2 — Visualization of the results of SHOE analysis and workflow on REACTOME and CellDesigner software tool of the GPX1 gene, principally the AML-1a transcription factor-binding motif. Note: Upper left, a full list of and the related motifs of arbutin is presented. In addition, the table shows the motif sequence, the consensus sequence, the similarity matching score and as well the pareto number.Upper right, alignment of the orthologous three species of the GPX1 promoter for the motif ALM-1a (H, human; M, mice; R, rat).Below left, the result table from SHOE is transferred to the program CellDesigner and the results are visualized in a hierarchical map.Below right, the program Reactome can also be referred to the data of SHOE and visualizes the pathway. [file Image_2.JPEG]

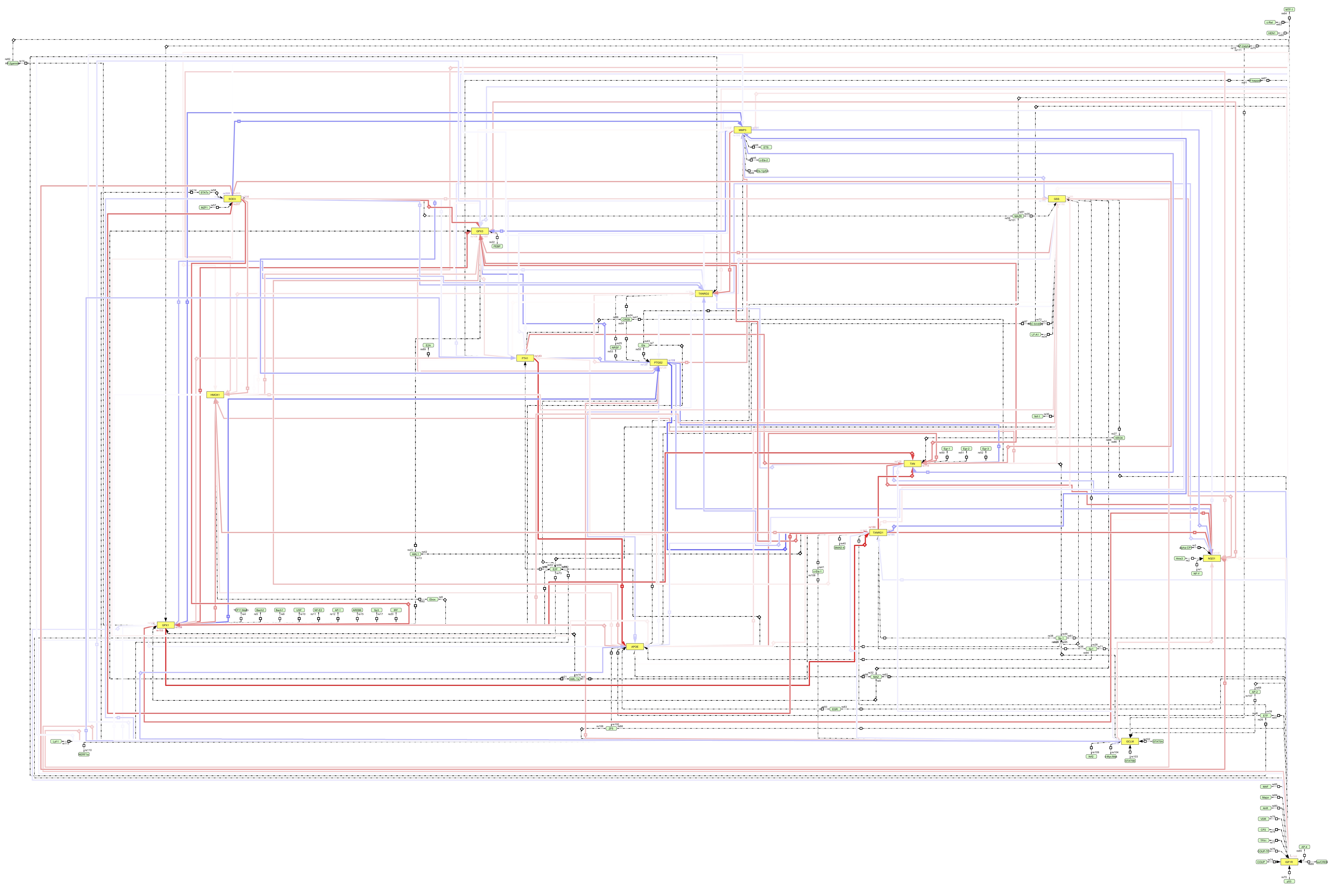

Supplement: Supplementary Figure 3 — Visualization of 15 orthologous genes using CellDesigner pathway editor. [file Image_3.JPEG]

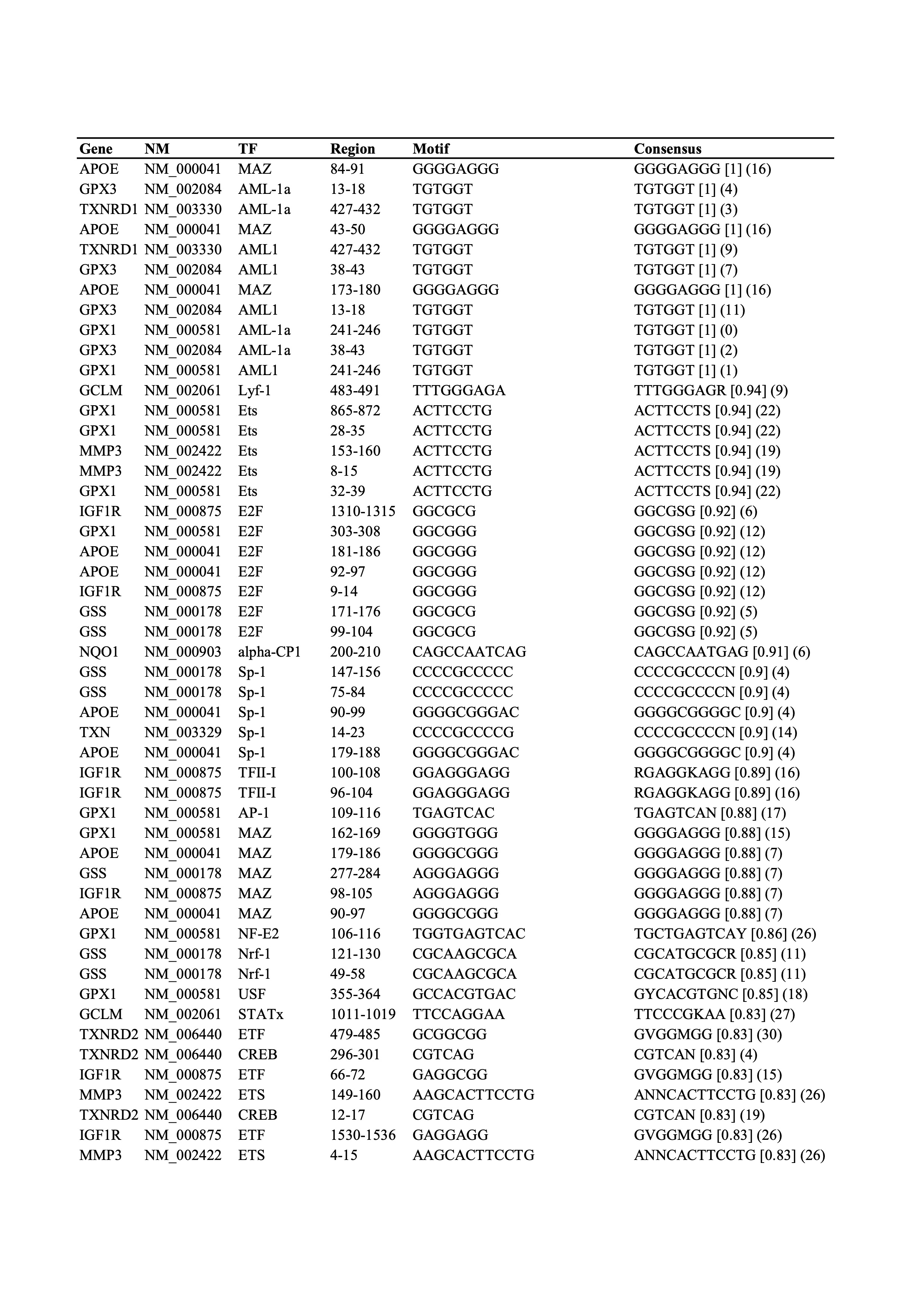

Supplement: Supplementary Table 1 — Oxidative stress gene with high expression change and a matching score to the consensus sequence over 70% are listed with the related transcription factor. Whereat the motifs from the oxidative stress gene are conserved.The complete list can be found on the SHOE site: http://ec2-54-150-223-65.ap-northeast-1.compute.amazonaws.comAbbreviation: NM, Refseq transcript number; TF, transcription factor.Note: The numbers behind the consensus presents the matching score between the motif and the consensus. In the second brackets, It is shown the pareto number. [file Image_4.JPEG]
